# Tipping the balance of cell death: alternative splicing as a source of MCL-1S in cancer

**DOI:** 10.1038/s41419-024-07307-z

**Published:** 2024-12-18

**Authors:** Mariusz L. Hartman

**Affiliations:** Department of Molecular Biology of Cancer, Medical University of Lodz, 6/8 Mazowiecka Street, 92-215 Lodz, Poland

**Keywords:** Cancer epigenetics, Cancer epigenetics

## Abstract

Apoptosis-regulating proteins from the B-cell lymphoma-2 (BCL-2) family are of continued interest as they represent promising targets for anti-cancer therapies. Myeloid cell leukemia-1 (MCL-1), which usually refers to the long isoform (MCL-1L) is frequently overexpressed in various types of cancer. However, *MCL1* pre-mRNA can also undergo alternative splicing through exon skipping to yield the short isoform, MCL-1S. Regarding its structure and function, MCL-1S corresponds to BCL-2 homology domain 3 (BH3)-only pro-apoptotic proteins in contrast to the pro-survival role of MCL-1L. As cancer cells are usually characterized by the high MCL-1L:MCL-1S ratio, several studies revealed that overexpression of MCL-1S may constitute a new therapeutic approach in cancer and presumably overcome resistance to currently available drugs. Switching the balance towards high levels of MCL-1S is feasible by using inhibitors of alternative splicing-regulating proteins and strategies directly interfering with *MCL1* pre-mRNA. Additionally, several compounds were shown to increase MCL-1S levels through unelucidated mechanisms, while diversely affecting the level of MCL-1L isoform. These mechanisms require detailed clarification as the balance between the long and short variants of MCL-1 can also contribute to mitochondrial hyperpolarization. In this respect, the role of MCL-1S in the regulation of apoptosis-unrelated events of the mitochondria physiology, including mitochondria fission and fusion also remains to be determined. In this review, the structure and function of MCL-1S isoform, and MCL-1S-targeting approaches are discussed.

## Facts

- MCL-1S is generated through alternative splicing, specifically via exon skipping
- MCL-1S acts as a pro-apoptotic protein by binding exclusively to MCL-1L
- MCL-1S can induce mitochondrial hyperpolarization
- Upregulating MCL-1S by interfering with splicing machinery induces apoptosis in cancer

## Open Questions

- Does MCL-1S play a role beyond apoptosis?
- How can MCL-1S regulate the mitochondria fission-fusion balance?
- How can MCL-1S be induced in cancer cells?
- Does baseline MCL-1S level have prognostic value in cancer patients?

## Introduction

Alternative splicing of pre-mRNA is a crucial mechanism of gene expression regulation at the post-transcriptional level that enriches cell transcriptome and promotes protein diversity. There are a few major types of alternative splicing events, including exon skipping, intron retention, selection of alternative splice sites or mutually exclusive exons, and selection of alternative promoters or polyadenylation [1]. Cancer cells are characterized by a number of splicing alterations resulting in affected transcriptomes that contribute to tumor development and progression, as well as response to therapy [2]. In this respect, different types of alternative splicing events constitute a source of various isoforms of proteins involved in the regulation of cell survival and death [3–6]. Myeloid cell leukemia-1 (MCL-1) was identified as an early induction gene during the differentiation of myeloblastic leukemia cells and extensively characterized as an apoptosis-regulating member of the B-cell lymphoma-2 (BCL-2) family of proteins [7]. The physiological roles of MCL-1 based on studies using genetic murine models and the implications of MCL-1 in the pathology and treatment of human diseases are still being elucidated [8, 9]. The long isoform of MCL-1 (MCL-1L) is of major interest due to its crucial pro-survival role. Accordingly, small-molecule inhibitors (BH3 mimetics) of MCL-1L imitating the function of pro-apoptotic BH3-only proteins demonstrated activity in different types of cancer when used alone or in combinations with other anti-cancer drugs [10–13], and they are under ongoing evaluations in clinical trials e.g., NCT04702425 and NCT03013998. However, adaptive resistance mechanisms to BH3 mimetics can develop [14–16], indicating that alternative approaches targeting the BCL-2 proteins are needed. In this review, the structure, function, and putative therapeutic inducers of MCL-1S, an understudied isoform of MCL-1 with pro-apoptotic activity are discussed in light of available literature.

## Structural comparison of short (MCL-1S) and long (MCL-1L) isoforms Of MCL-1

**Fig. 1:**
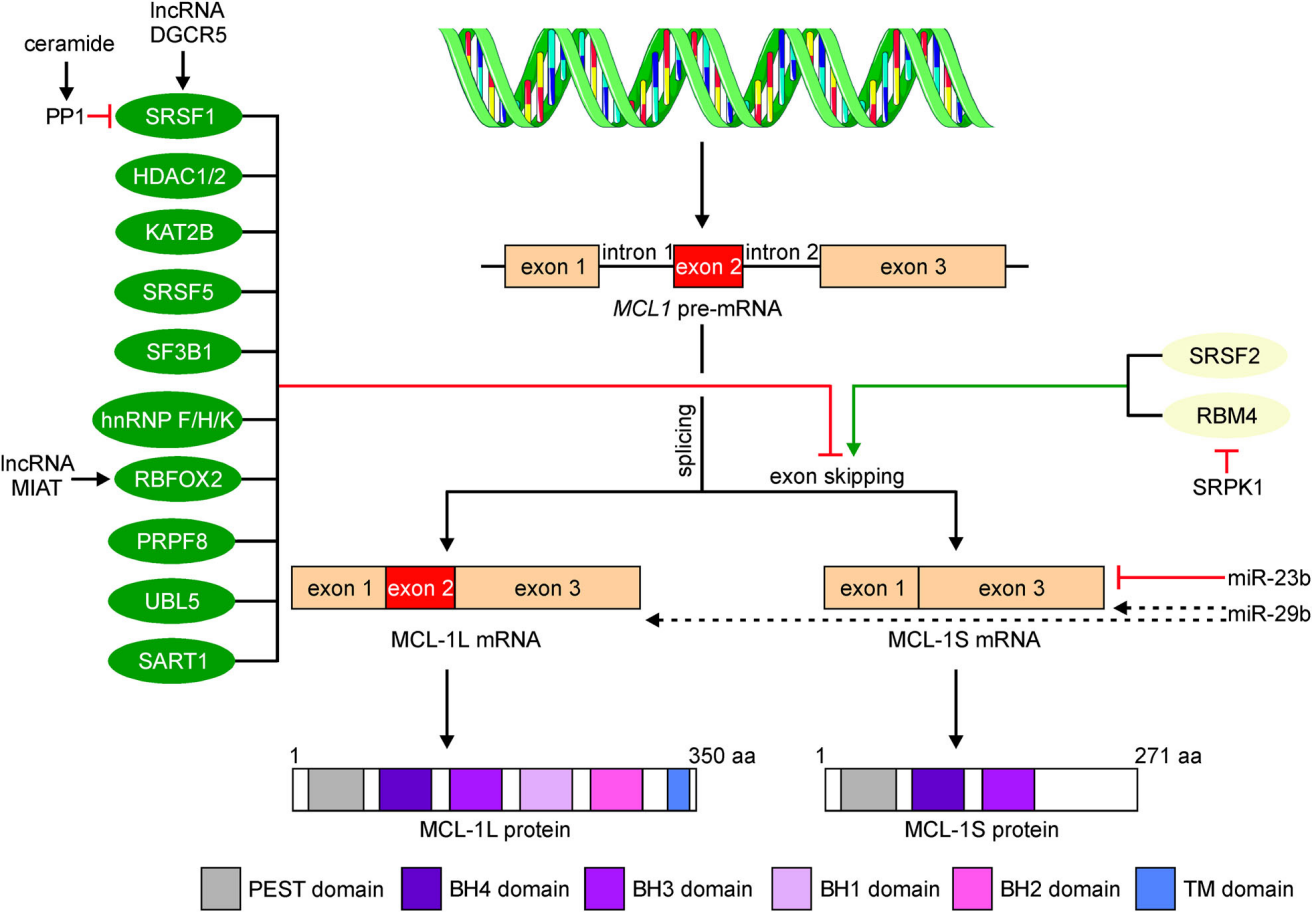
Regulation of alternative splicing of the *MCL1* pre-mRNA. Negative (left side) and positive (right side) regulators of the splicing switch leading to MCL-1S expression are indicated. Although MCL-1ES isoform can also be generated through alternative splicing, only MCL-1S and MCL-1L are included as they are in the scope of this review. Additional regulators such as lncRNA and miR are shown. An ambiguous influence of miR-29b on the MCL-1L and MCL-1S mRNAs is demonstrated by dotted lines. BH domain, BCL-2 homology domain; DGCR5, DiGeorge syndrome critical region gene 5; HDAC, histone deacetylase; hnRNPs, heterogeneous nuclear ribonucleoproteins; KAT2B, lysine acetyltransferase 2B; lncRNA, long non-coding RNA; MIAT, myocardial infarction–associated transcript; miR, micro RNA; PEST domain, polypeptide sequences enriched in proline (P), glutamic acid (E), serine (S) and threonine (T); PP1, protein phosphatase 1; PRPF8, pre-mRNA processing factor 8; RBFOX2, RNA-binding fox-1 homolog 2; RBM, RNA-binding motif; SART1, spliceosome associated factor 1; SF3B1, splicing factor 3B1; SRSF, serine and arginine-rich splicing factor; UBL5, ubiquitin-like protein 5.

The *MCL1* gene is composed of three exons undergoing alternative splicing to generate three types of mRNA transcripts: long (MCL-1L), short (MCL-1S), and extra short (MCL-1ES) [17]. A short splicing variant of the MCL-1 mRNA was first identified in 2000 in the human placenta and myelogenous leukemia cell line [18], primary human hematopoietic cells, macrophages, neutrophils, T-cell leukemia cells, and lung carcinoma cell line [19]. MCL-1S (also known as MCL-1_ΔTM_) transcript is a product of exon 2 skipping during processing of the *MCL1* pre-mRNA by deletion of 248 nucleotides [18, 19]. As a consequence, MCL-1S of 271-amino acid in length lacks BCL-2 homology (BH) domains (BH1 and BH2) and transmembrane (TM) domain compared with MCL-1L isoform of 350 amino acid residues in length (Fig. 1) [18, 19]. While the first 229 amino acid residues of MCL-1S variant are identical to MCL-1L, a shift in the reading frame leads to a synthesis of a unique C-terminal fragment of MCL-1S containing six cysteine residues and multiple residues of basic amino acids [18].

## Regulation of MCL-1S expression and activity

### Alternative splicing as a source of the MCL-1S mRNA abundance

The MCL-1S transcript is co-expressed with MCL-1L, although the levels of MCL-1S are lower than those of MCL-1L as demonstrated in different biological systems [18–22]. This results from common transcriptional regulation of the *MCL1* gene while involving specific alternative splicing events determining the level of MCL-1S within the cell. Alternative splicing is meticulously controlled and regulated by the interplay between *trans*-splicing factors and *cis*-responsive elements within the regulated exons and involves different classes of RNA-binding proteins (RBPs) including serine and arginine-rich (SR) proteins, heterogeneous nuclear ribonucleoproteins (hnRNPs), spliceosome components, and RNA-binding motif (RBM) proteins [23–26]. The contribution of several types of RBPs to the regulation of alternative splicing of *MCL1* pre-mRNA was demonstrated (Fig. 1). Specific binding site for serine and arginine-rich splicing factor 1 (SRSF1) was identified in the second exon of *MCL1* pre-mRNA [27]. Inhibition of SRSF1 resulted in an elevated level of MCL-1S transcript and protein in breast cancer, choriocarcinoma [28], colorectal cancer cells [29], and human primary neurons exposed to ethanol [30]. Mechanistically, it was shown that this effect resulted from an increased level of ceramide and the activation of protein phosphatase 1 (PP1), which inhibited the nuclear translocation of SRSF1 in ethanol-exposed neurons [31]. These observations are consistent with a previous study showing that the induction of MCL-1S correlated with increased activity of PP1 [32]. It was also shown that SRSF1 interacted with histone deacetylases (HDACs), HDAC1 and HDAC2, which were recruited to the *MCL1* pre-mRNA along with lysine acetyltransferase 2B (KAT2B) [29]. HDAC availability was involved in the regulation of exon 2 exclusion as increased MCL-1S levels were demonstrated upon either HDAC knockdown or pharmacological inhibition [29]. In addition, attenuation of SRSF5 was associated with an increased level of MCL-1S isoform, although this effect was cell type-specific [28]. The hnRNP K and members of the hnRNP F/H family were identified as splicing enhancers promoting the inclusion of exon 2 leading to the generation of MCL-1L. A direct mechanism of hnRNP K and hnRNP F/H activity in the *MCL1* pre-mRNA splicing was confirmed by RNA immunoprecipitation, and a specific binding site for the hnRNP F/H protein was identified using the *MCL1* minigene construct. This binding site acted as an intronic splicing enhancer site, and its location of 24 bp from the 5ʹ splice site was typical for a G tract involved in enhancing splicing [27]. Conversely, downregulation of hnRNP K and hnRNP F/H resulted in a splicing switch towards the MCL-1S, and a substantial increase in the level of MCL-1S could be achieved through double and triple knockdowns of these RBPs [27]. The role of splicing factor 3B1 (SF3B1) in the regulation of an alternative pre-mRNA splicing sustaining a high MCL-1L:MCL-1S ratio was also shown [33]. Inhibition of SF3B1 resultantly upregulated MCL-1S transcript and protein while reducing the levels of MCL-1L [34, 35]. Increased splicing towards MCL1-S following the knockdown of spliceosome complex members: pre-mRNA processing factor 8 (PRPF8), ubiquitin-like protein 5 (UBL5), and spliceosome associated factor 1 (SART1) was demonstrated, and was associated with an elevated MCL1-S:MCL1-L ratio [35]. In turn, the depletion of SRSF2 was followed by decreased levels of several pro-apoptotic proteins, including MCL-1S in renal cancer cells [36]. Elevated SRPK1 levels induced the cytoplasmic accumulation of phosphorylated RBM4, which prevented from pro-apoptotic activity of RBM4 associated with the generation of the MCL-1S transcript. Indeed, either downregulation of SRPK1 or overexpression of RBM4 was associated with increased levels of MCL-1S [37].

In addition to proteins directly involved in alternative splicing, a few non-coding RNA can affect the MCL-1S mRNA level, while some of these regulators also interfere with alternative splicing machinery. Long non-coding RNA (lncRNA) DiGeorge syndrome critical region gene 5 (DGCR5) was associated with the stabilization of SRSF1, which resulted in an increased level of MCL-1L in esophageal squamous cell carcinoma [38]. Myocardial infarction–associated transcript (MIAT) was shown to bind and stabilize RNA-binding fox-1 homolog 2 (RBFOX2) protein, thereby promoting RBFOX2-induced upregulation of MCL-1L and reduction of pro-apoptotic MCL-1S [39]. In addition, miR-23b was evidenced to decrease the level of MCL-1S mRNA in lung cancer cells while not affecting MCL-1L isoform [40]. In combination with dihydroartemisinin, miR-29b increased the level of MCL-1S in cholangiocarcinoma cells [41], although the effect of miR-29b on the MCL-1S level was cell type-specific as shown in another study [42]. It was also demonstrated that miR-29b predominantly increased the MCL-1L isoform at both the mRNA and protein levels in intestinal fibroblasts [22]. As a binding site for miR-29b was predicted within 3’UTR, which is identical for all MCL-1 isoforms [22], these studies suggest that the influence of miR-29b on the level of specific MCL-1 isoforms could be different in normal and cancer cells.

### Regulation of the MCL-1S protein level and activity

**Fig. 2:**
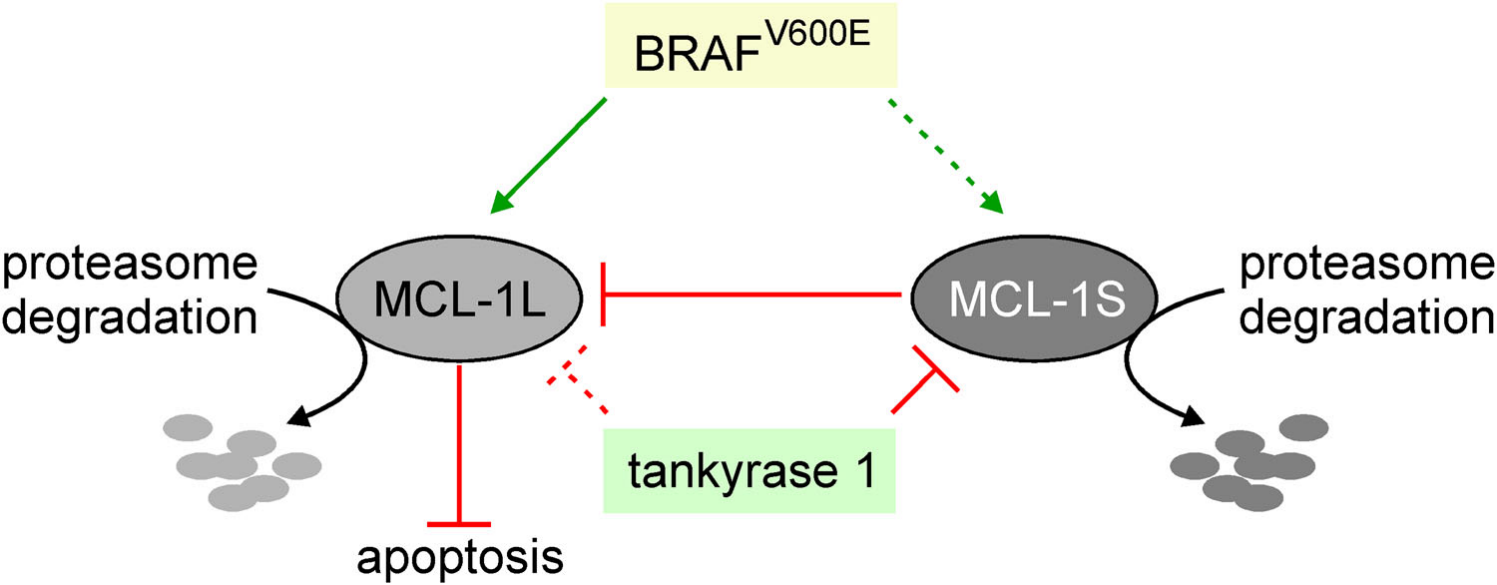
Regulation of the level and activity of MCL-1S. MCL-1S shares common regulators with MCL-1L such as BRAF^V600E^ and tankyrase 1, although MCL-1 isoform-specific influence was partly found. Both isoforms of MCL-1 are subjected to proteasomal degradation. MCL-1L is the exclusive binding partner of MCL-1S, indicating that MCL-1S promotes apoptosis by antagonizing anti-apoptotic function of MCL-1L.

Only a few proteins specifically involved in the regulation of the MCL-1S protein levels were found (Fig. 2). The yeast two-hybrid screening revealed an interaction between MCL-1S/MCL-1L and tankyrase 1, a protein with the poly(ADP-ribose) polymerase activity [43]. Mechanistically, it was demonstrated that the first ten ankyrin repeats were involved in the interaction between tankyrase 1 and a short 25-amino acid N-terminal fragment of MCL-1 [43]. It was also shown that tankyrase 1 antagonized MCL-1S and MCL-1L activities by reducing the protein levels of both isoforms of MCL-1 in an ADP-ribosylation-independent manner. In turn, overexpression of MCL-1 suppressed the ADP-ribosylation of the telomeric repeat binding factor 1 (TRF1), another tankyrase 1-interacting protein [43]. In lung adenocarcinoma cells, the antisense oligonucleotide of tankyrase increased MCL-1S protein levels more pronouncedly than MCL-1L [44]. In addition, elevated protein levels of MCL-1S and MCL-1L were found in BRAF^V600E^ melanoma cell lines, and in *BRAF* wild-type cells transfected with BRAF^V600E^ construct [45]. Inhibition of BRAF^V600E^ activity, however, did not affect the MCL-1S level [45]. In turn, both MCL-1S and MCL-1L were upregulated upon inhibition of proteasome activity [46].

## Binding affinity of MCL-1S to other proteins from the BCL-2 family

The mutual interactions between specific proteins from the BCL-2 family are key mechanisms regulating their activity. MCL-1S, unlike MCL-1L, did not interact with other pro-apoptotic BCL-2-related proteins in the yeast two-hybrid system [18]. In turn, it was shown that MCL-1S dimerized exclusively with MCL-1L and coprecipitated with MCL-1L in transfected mammalian cells [18]. This heterodimerization appeared stoichiometric as comparable amounts of MCL-1L and MCL-1S proteins were coprecipitated [18]. It was later validated by showing that the stabilized BH3 α-helices of MCL-1S selectively interacted with the hydrophobic groove of MCL-1L but not that of other anti-apoptotic proteins [47]. Since MCL-1S lacks the TM domain, its interaction with MCL-1L may enable MCL-1S to localize in the mitochondrial membrane, although MCL-1S was also observed in the nuclei [48]. Overexpression of MCL-1S was associated with dose-dependent apoptosis in CHO cells that could be prevented by caspase inhibition and concurrent overexpression of MCL-1L [18]. Similarly, overexpression of MCL-1S efficiently induced apoptosis in basal cell carcinoma (BCC) cells [48]. It was also demonstrated that exposure of neurons to ethanol was accompanied by favoring MCL-1S splicing [30], while ectopic expression of MCL-1L was sufficient to protect these cells from alcohol exposure-induced apoptosis [49]. Altogether, this indicates that the MCL-1S variant represents a pro-apoptotic BH3-only protein, and its activity is antagonized by MCL-1L creating a ‘rheostat model’ that may affect cell survival and death (Fig. 2). In this respect, MCL-1S is the only known splice variant within the BCL-2 family of proteins that can dimerize exclusively with another variant of the same protein [50].

## Manipulation with alternative splicing machinery to promote MCL-1S expression

**Fig. 3:**
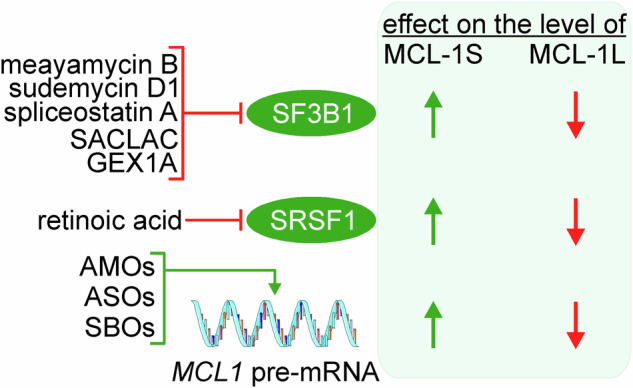
Potential therapeutic approaches influencing alternative splicing machinery involved in the processing of the *MCL1* pre-mRNA. Experimentally evidenced targets for each compound are shown, although further research is necessary to more broadly determine their mechanisms of action. AMOs, ASOs, and SBOs directly modulate the alternative splicing of *MCL1* pre-mRNA to promote the generation of MCL-1S mRNA. The green arrow indicates the upregulation of MCL-1S, and the red arrow indicates the downregulation of MCL-1L, which is associated with the induction of apoptosis. AMOs, antisense morpholino oligonucleotides; ASOs, splice-switching antisense oligonucleotides; SBOs, steric-blocking oligonucleotides.

Considering the opposite functions of MCL-1L and MCL-1S isoforms, it is expected that their levels could determine the clinical outcome of cancer patients. A switch in the alternative splicing of MCL-1 was shown in breast cancer, which was characterized by upregulation of MCL-1L compared to the increased level of MCL-1S in normal tissue [51]. Accordingly, it was also shown that the levels of MCL-1S were lower in BCC cells than in normal primary keratinocytes [48]. Patients with MCL-1S^low^ oral cancers exhibited significantly worse overall survival compared to MCL-1S^high^ patients [21]. In addition, a high ratio of MCL-1L:MCL-1S showed a significant correlation with the poor overall survival of these patients [21]. As various mechanisms of switch towards elevated levels of MCL-1L were shown in cancer cells [38, 40, 52], targeting the alternative splicing machinery to promote MCL-1S expression can be exploited to improve the efficacy of anti-cancer drugs (Fig. 3). It was evidenced that retinoic acid, which induced the differentiation of HL60 cells into granulocytic cells, increased the MCL-1S protein level by activating protein kinase A (PKA), which was associated with phosphorylation and inactivation of SFSR1 in the nucleus [53]. SACLAC, (N-[(2S,3 R)-1,3-dihydroxyoctadecan-2-yl]2-chloroacetamide), an inhibitor of acid ceramidase reduced the SF3B1 and increased MCL-1S levels in acute myeloid leukemia cell lines, while not affecting SRSF1 level [54]. The mechanism of SACLAC activity was confirmed by the knockdown of acid ceramidase and exogenous C16-ceramide supplementation that induced similar changes in SF3B1 level and MCL-1S level [54]. GEX1A (herboxidiene), a splicing modulator interfering with SF3B1 and originally isolated from *Streptomyces sp*., selectively induced alternative splicing of MCL-1 leading to the increase in MCL-1S level and apoptosis in leukemic cells both in vitro and in vivo [55]. There are also other inhibitors of SF3B1, although their influence on MCL-1S level was insufficiently investigated. It was shown that inhibition of SF3B1 by meayamycin B in combination with ABT-737, an inhibitor of BCL-2, BCL-XL, and BCL-w, triggered apoptosis in non-small cell lung cancer cells insensitive to ABT-737 used alone [34]. Mechanistically, meayamycin B induced a preferential splicing of the *MCL1* pre-mRNA leading to a higher MCL-1S:MCL-1L ratio than in control cells [34]. Notably, since SF3B1 is also a *trans*-acting splicing factor for the BCL-XL/BCL-XS as shown in HeLa cells [33], meayamycin B did not significantly increase BCL-XS in lung cancer cells [34]. This suggests that meayamycin B was selective for MCL-1, although it could be cell type-specific or related to differences in strategies applied to inhibit SF3B1 as either siRNA [33] or chemical approach [34] were used. Spliceostatin A also increased the MCL-1S:MCL-1L ratio and induced apoptosis cooperatively with ABT-737 in chronic lymphocytic leukemia (CLL) cells, but the pro-apoptotic effect of the drug combination resulted from the accompanying down-regulation of MCL-1L [56]. Sudemycin D1, another inhibitor of SF3B1, modulated the splicing of several genes including MCL-1, both in *SF3B1*-mutated and wild-type CLL cells, although this effect was validated exclusively at the mRNA level [57].

While the pharmacological strategies targeting upstream regulators of the *MCL1* pre-mRNA alternative splicing should be regarded with caution due to the potential broad range of the regulated proteins, other strategies directly interfering with the *MCL1* pre-mRNA were also developed. The antisense morpholino oligonucleotides (AMOs) were used to knock out exon 2 within the *MCL1* pre-mRNA. These AMOs targeted the 3’ acceptor and 5’ donor splice site boundaries flanking exon 2 to join exons 1 and 3 in the *MCL1* pre-mRNA. As a result, the level of pro-apoptotic MCL-1S was increased in parallel with the downregulation of MCL-1L, which consequently induced apoptosis in BCC cells and gastric adenocarcinoma cells [48]. Steric-blocking oligonucleotides (SBOs) used to shift splicing from MCL-1L to MCL-1S efficiently downregulated MCL-1L, increased MCL-1S level, and promoted cancer cell apoptosis in a dose-dependent manner that was additionally confirmed in mouse xenotransplant models [52]. MCL-1-specific SBOs increasing the MCL-1S level also reversed the cytoprotective effect of RBFOX2 overexpression on H_2_O_2_-induced injury in pheochromocytoma cells [39]. A concurrent elevated synthesis of MCL-1S and reduction of MCL-1L was also demonstrated using splice-switching antisense oligonucleotides (ASOs) in HeLa cells [58]. Switching to elevated MCL-1S level was associated with enhanced sensitivity of cancer cells to agents inducing Ca^2+^-dependent apoptosis. It was also demonstrated that the MCL-1 ASOs induced hyperpolarization and increased calcium concentration in the mitochondria. In this respect, a high MCL-1S:MCL-1L ratio correlated with a dynamin-related protein (DRP1)-dependent hyperfusion of the mitochondrial network [58]. Taking into account that the mitochondria dynamics involving fission-fusion balance can diversely determine the cell fate by leading to either induction of apoptosis or cell adaptation to various stressors [59–61], it remains to be more broadly investigated whether the influence of MCL-1S on the mitochondria morphology has an exclusively apoptosis-related outcome and whether MCL-1S plays additional apoptosis-unrelated roles associated with the regulation of mitochondria physiology.

## Other potential therapeutic approaches influencing MCL-1S levels in cancer cells

Several other drugs and compounds with anticancer activity were demonstrated to affect the MCL-1S level, however further research is needed to investigate their detailed modes of action and determine to what extent they are selective towards MCL-1S/L (Table 1). M2I-1, the first small-molecule inhibitor of MAD2 that disrupts the CDC20-MAD2 was shown to induce the MCL-1S cooperatively with nocodazole, which causes the depolymerization of microtubules [62]. Ectopically expressed MCL-1S sensitized MCL-1S^low^ breast cancer cells to apoptosis induced by M2I-1 and nocodazole used in combination [62]. MCL-1S was capable of translocation to the nuclear compartment, while overexpression of MCL-1S or knockdown of MCL-1L led to cell arrest in the G1 phase of the cell cycle and was associated with the accumulation of DNA damage [63]. This suggested a cell cycle-associated role of MCL-1S. Importantly, S63845, a BH3 mimetic targeting MCL-1L did not cause the same response, suggesting that either MCL-1L exhibited its cell cycle-associated role independently of the BH3-binding groove or S63845 failed to efficiently antagonize the nuclear fraction of MCL-1L due to the abundance of cytoplasmic MCL-1L pool [63]. This, however, requires further research as S63845 was shown to increase γ-H2AX levels in melanoma cells [11]. Genotoxic drugs such as cisplatin, doxorubicin, and etoposide did not affect MCL-1S level in HEK293T cells, although anti-mitotic agent monastrol caused accumulation of MCL-1S mRNA without increasing the protein level [63].

**Table 1:** Drugs and factors affecting the level of MCL-1S isoform in different types of cancer cells by undetermined modes of action.

Cell type	Cell line	Drugs/factors increasing MCL-1S level	Effect on the MCL-1L level	Ref.
Acute myeloid leukemia	HL60	YM155	Downregulation	[73]
Acute myeloid leukemia and chronic myeloid leukemia	HL60, K562	α-tomatine	No substantial changes	[71]
Acute T-cell leukemia	Jurkat	5-fluorouracil	Downregulation	[72]
Breast cancer	MCF-7	Undecylprodigiosin	Unaltered	[67]
Breast cancer	MCF-7	*N*-methyl bis(indolyl)hydrazide-hydrazone derivative	Unaltered	[68]
Breast cancer	MDA-MB-231	Ethyl acetate fraction of *Stylissa carteri*	ND	[69]
Cervical cancer	HeLa	M2I-1 + nocodazole	Slight upregulation	[62]
Cholangiocarcinoma	QBC939	Dihydroartemisinin	No substantial changes	[64]
Cholangiocarcinoma	HUCCT-1, FRH0201	Dihydroartemisinin + miR-29b	Downregulation	[41]
Multiple myeloma	MM.1S	SDX-101	No substantial changes	[70]
Prostate cancer	PC-3	Caseamembrin C	Slight upregulation	[65]
Prostate cancer	DU145	Isoangustone A	No substantial changes	[66]
Prostate cancer	PC-3	EGCG and ibuprofen	Downregulation	[32]
Tongue squamous cell carcinoma	AW8507	Irradiation	Upregulation	[74]

Cell type	Cell line	Drugs decreasing MCL-1S level	Effect on the MCL-1L level	Ref.
Head and neck squamous cell carcinoma	CAL27, UM-SCC1	Curcumin (liposome-encapsulated)	Downregulation	[76]

*ND* not determined.

The effect of each drug/factor on the level of long isoform of MCL-1 (MCL-1L) is included for comparison if it was assessed concomitantly in the research.

Several studies only reported changes in the MCL-1S level in response to particular drugs or factors, and therefore the validation of the direct contribution of MCL-1S to the induction of apoptosis under these conditions is required. These drugs/factors can, however, be considered in further research as putative inducers of MCL-1S. In this respect, dihydroartemisinin, a sesquiterpene lactone extracted from *Artemisia annua*, induced apoptosis in the cholangiocarcinoma cells by upregulating MCL-1S and decreasing MCL-1L:MCL-1S ratio [64]. Overexpression of miR-29b in cholangiocarcinoma cells followed by exposure to dihydroartemisinin demonstrated increased pro-apoptotic effect compared to dihydroartemisinin alone that was associated with the concomitant upregulation of MCL-1S and downregulation of MCL-1L, however, the influence of miR-29b used alone was not assessed [41]. Caseamembrin C, a clerodane diterpene isolated from *Casearia membranacea*, increased the level of MCL-1S protein in prostate cancer cells that accompanied caseamembrin C-induced cell death [65]. Epigallocatechin-3-gallate (EGCG) and ibuprofen synergistically induced apoptosis in prostate cancer cells *via* regulating alternative splicing of MCL-1 and BCL-X, specifically by down-regulating the mRNA levels of anti-apoptotic isoforms (MCL-1L and BCL-XL) with a concomitant increase in the transcript levels of pro-apoptotic MCL-1S and BCL-XS, respectively [32]. This effect was, however, cell line-specific [32]. Concentration-dependent upregulation of MCL-1S protein was shown in prostate cancer cells exposed to hexane/ethanol extract of *Glycyrrhiza uralensis* (HEGU), while isoangustone A was identified as an active compound in HEGU [66]. Undecylprodigiosin, a bioactive metabolite produced by *Streptomyces* and *Serratia*, induced apoptosis in breast cancer cells that was accompanied by a slight upregulation of MCL-1S [67]. A novel *N*-methyl bis(indolyl)hydrazide-hydrazone derivative [68] and ethyl acetate fraction of marine sponge *Stylissa carteri* used alone and combined with paclitaxel substantially increased MCL-1S protein level in breast cancer cells [69]. It was also demonstrated that SDX-101, the *R*-enantiomer of etodolac, increased MCL-1S level while enhancing the pro-apoptotic effect of dexamethasone in multiple myeloma cells [70]. In leukemia cells, α-tomatine had a significant cytotoxic effect accompanied by upregulation of MCL-1S [71]. 5-fluorouracil induced apoptosis in Jurkat cells that was associated with the downregulation of MCL-1L and the induction of pro-apoptotic MCL-1S in a time-dependent manner [72]. YM155, a small molecule inhibitor of survivin, upregulated MCL-1S level and induced apoptosis in acute myeloid leukemia HL60 cells, but not in U937 cells [73]. In addition, MCL-1S level increased after irradiation of tongue squamous cell carcinoma cells [74]. MCL-1S was rapidly accumulated in the mitochondrial fraction of colon cancer cells exposed to MG132, a proteasome inhibitor [46]. This effect was similar in both wild-type cells and their *BAX* or *TP53* knock-out counterparts [46]. A transient increase in MCL-1S protein levels was also found in cells exposed to bortezomib, another inhibitor of proteasome activity. However, accumulation of MCL-1S was not observed in bortezomib-resistant mesothelioma cells [75]. In turn, MCL-1S and MCL-1L protein levels were diminished by liposome-encapsulated curcumin in head and neck squamous cell carcinoma cells [76].

## Conclusions and future perspectives

In this review, the available knowledge on the structure and function of MCL-1S, as well as potential targeting options for increasing the levels of this pro-apoptotic protein were summarized. While the essential role of MCL-1L in the resistance of multiple types of cancer to apoptosis is recognized and the development of novel MCL-1L inhibitors is ongoing [77–79], influencing the level of MCL-1S is much less investigated. Induced overexpression of MCL-1S, especially concomitant with downregulation of MCL-1L, is expected to increase apoptosis or sensitize cells to induction of apoptosis by other drugs. Distinct approaches regulating alternative splicing of *MCL1* pre-mRNA by switching to exon 2 skipping to produce pro-apoptotic MCL-1S can have therapeutic potential. This is supported by the growing availability of SF3B1 inhibitors [80, 81] and the current research on splice-switching antisense oligonucleotides [82–85], antisense morpholino oligonucleotides [86], and steric-blocking oligonucleotides [87] targeting various mRNAs in different types of disorders. It, however, remains to be determined whether and to what extent they can be relevant to promote MCL-1S expression in cancer cells, and how they concomitantly modulate the level of anti-apoptotic MCL-1L. Another crucial issue is the monitoring of the expression of two MCL-1 isoforms both before MCL-1-targeted interventions and during the course of apoptosis. As the contributions of cancer-specific isoforms generated by alternative splicing events are still largely unknown [2], this is especially important considering the putative role of MCL-1S in the modulation of mitochondria fission-fusion balance. In this respect, further studies using comprehensive gene perturbation approaches and orthogonal methods [88, 89] are crucial to more broadly elucidate the role of MCL-1S in apoptosis and apoptosis-unrelated events to efficiently target MCL-1S in cancer.
